# Collective Attention and Stock Prices: Evidence from Google Trends Data on Standard and Poor's 100

**DOI:** 10.1371/journal.pone.0135311

**Published:** 2015-08-10

**Authors:** Raphael H. Heiberger

**Affiliations:** Institute for Sociology, University of Bremen, Bremen, Germany; East China University of Science and Technology, CHINA

## Abstract

Today´s connected world allows people to gather information in shorter intervals than ever before, widely monitored by massive online data sources. As a dramatic economic event, recent financial crisis increased public interest for large companies considerably. In this paper, we exploit this change in information gathering behavior by utilizing Google query volumes as a "bad news" indicator for each corporation listed in the Standard and Poor´s 100 index. Our results provide not only an investment strategy that gains particularly in times of financial turmoil and extensive losses by other market participants, but reveal new sectoral patterns between mass online behavior and (bearish) stock market movements. Based on collective attention shifts in search queries for individual companies, hence, these findings can help to identify early warning signs of financial systemic risk. However, our disaggregated data also illustrate the need for further efforts to understand the influence of collective attention shifts on financial behavior in times of *regular* market activities with less tremendous changes in search volumes.

## Introduction

In the past decade connections of people all around the globe have dramatically increased due to technological innovations related to the internet. The ongoing worldwide computerization and integration provides great opportunities for scientists to enhance our understanding of the complex systems in which humans live today. Increasing availability of massive social media data abets efforts trying to explain collective behavior with methods stemming from the natural sciences [1–7], allowing to transfer knowledge about mechanisms already found in, for instance, complex ecological systems [8–10].

Given the impact of the recent financial crisis on economic wealth, political decisions and personal fortunes, special interest bestow researchers upon patterns in modern financial markets [11–23]. For an approximation of collective financial behavior diverse online sources have already been used that yield complementary results: strong correlations are reported between trading volumes of securities and the frequency brand names appear on Twitter [24], and the number of daily search queries on Yahoo [25], respectively. Editing activity in Wikipedia is linked to critical events in the near future [26], and the text content of daily tweets is analyzed in respect to its mood and found to be predictive of changes in the values of the Dow Jones Industrial Average [27]. Moreover, the Bitcoin crypto-currency and its price dynamics have been shown to exhibit, besides more fundamental and technical drivers [28], strong relationships with the number of new users, Wikipedia page views and search queries provided by Google Trends data [29,30]. The latter, publicly available service seems to be especially fruitful for scientists to comprehend collective financial behavior. Therein, Google provides access to aggregated information on the volume of queries for specific search terms over time. Although mostly capturing the attention of uninformed investors [31], those online search query data have delivered useful information to predict trading volumes [32], to diversify portfolio risks [33], and to quantify trading behavior with given keywords [34] or with semantic topics derived by a latent Dirichlet allocation (“topic modelling") of Wikipedia articles [35].

To explain collective financial actions, it is helpful to remind Herbert Simon´s [36] famous notion that decisions of economic actors start with the gathering of information, yet, that the attention of those actors is rather limited compared to the amount of available information. His observation stems from the 1950s, but seems to be more valid than ever in modern societies. Real-time information supply from countless online sources makes selection processes for investors increasingly important and for scientists an even richer research area. However, a series of problems clouds the possibilities of social media data [37–39]. In addition to more general issues of adequate methodological standards for analyzing large social media data discussed by Ruths and Pfeffer [40], we can add, in accordance with Sun and others [41], that the cited studies concerned with collective human behavior and financial markets mainly focus on the prediction of composite indices. In contrast, the influence of collective attention shifts on individual stock price movements is so far a widely unexplored question.

This paper tries to fill this gap by investigating not only aggregate compositions but individual stock prices and their connection to firm-specific volumes of Google search queries. The disaggregated data set allows the examination of the direct relationship between stock prices and company search volumes on different levels of aggregation. By including company-level information about sectoral affiliations we can study how diverse ways of doing business in different branches inspire different information gathering strategies. Moreover, we can also investigate individual company performances. The connection between stock markets and Google search volume becomes evident on all levels during recent financial turmoil; a period, in which returns of a Google Trend based strategy by far exceed average market developments. Our findings are consistent with the intuition that recessions are highly suitable for market predictions made by collective behavior indicators, since such downturns draw mass attention to economic issues in general and therein to the most affected business sectors in particular.

## Results

For our analysis we gathered the search volumes provided by Google Trends for all companies listed in the Standard & Poor´s 100 (SPY) in August, 2014 (see Material section for further details and S1 File for the actual data) [42]. The SPY index composition is based on one hundred large and well established “blue chips” and represents ten major economic branches defined by the Global Industry Classification Standard [43]. We used the full corporate name in combination with “company” as search term to avoid semantic ambiguities. The scores produced by Google Trends consist of the volume of each search query relative to the total number of searches carried out at each point in time. The results are reported weekly and the subsequent data set was collected for the period between 4 January, 2004 and 4 January, 2014. For five companies we could not find any Google Trends scores with the described search terms, they are excluded from further analysis (an overview of the missing data is provided in S2 File). The following results are therefore based on the remaining 95 stocks, and a stricter sample (yielding similar results) is discussed in the Material section.

In order to identify shifts in collective information retrieval behavior we calculate relative changes in search volumes for each stock: Δ*n*_*i*_ (*t*,Δ*t*) = *n*_*i*_ (t) − *N*_*i*_ (*t*– 1,Δ*t*), with, *N*_*i*_ (*t*– 1,Δ*t*) = (*n*_*i*_ (*t*– 1) + *n*_*i*_ (t– 2)+…+*n*_*i*_ (*t* − Δ*t*)) / Δ*t*, where *n*_*i*_ is the relative search volume for stock *i* and Δ*t* is set to three weeks, as done by Preis and colleagues [34]. To analyze the average change for each week over all company search queries we use $\langle \Delta n\rangle \left(t,\;\Delta t\right)=\sum_{i}^{100}\Delta n_{i}\left(t,\;\Delta t\right)/100$ and $\langle \Delta n_{S}\rangle \left(t,\;\Delta t\right)=\sum_{i}^{s}\Delta n_{i}\left(t,\;\Delta t\right)/S$ for companies belonging to sector *S*, respectively.

**Fig 1:**
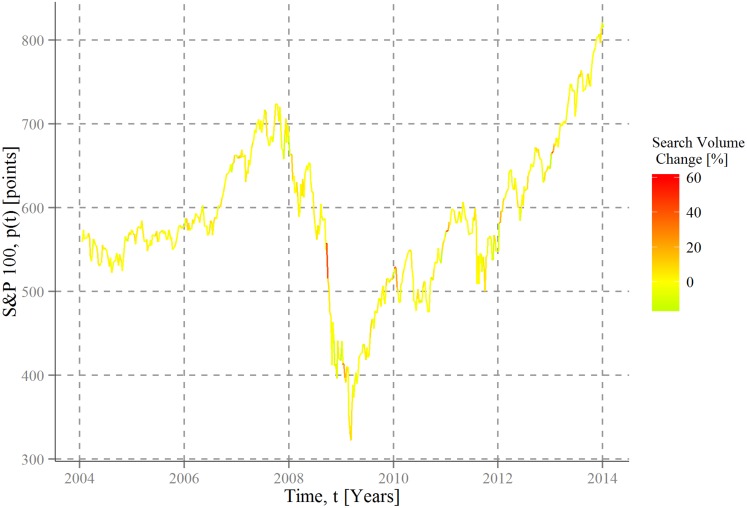
Company search volumes and stock market movement. Closing prices *p*(*t*) of the Standard and Poor´s 100 index are plotted as a function of time for the first day of every week in the period between 4 January, 2004 and 4 January, 2014. The subsequent curve is dyed by a color code corresponding to the average change of search volumes for each of the Standard and Poor´s 100 companies. Red sections indicate increasing collective attention in terms of search volumes and green parts illustrate declining number of search queries. The average for each week over all company search queries is calculated by $\langle \Delta n\rangle \left(t,\;\Delta t\right)=\sum_{i}^{100}\Delta n_{i}\left(t,\;\Delta t\right)/100$, with Δ*t* being 3 weeks. Please see S3 File for different time windows of Δ*t*.

Fig 1 shows for company-based queries of the SPY components in the period 2004–2014 that collective information gathering behavior is especially high in bearish markets during the collapse of Lehman Brothers and, to a lower degree, during the unfolding Euro-crisis in the beginning of 2010. Large attention increases occur only during those slumps, whereas declining numbers of search queries are more likely in rising markets.

**Fig 2:**
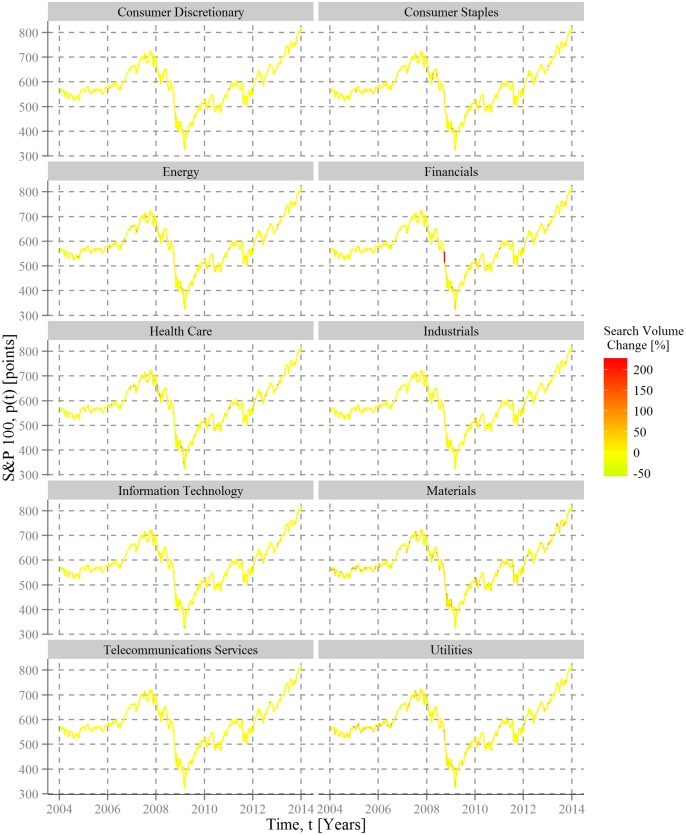
Sectoral search volumes and stock market movement. For each sector of the companies contained in the Standard and Poor´s 100 the development of closing prices *p*(*t*) of the index is drawn as a function of time for the first day of every week in the period between 4 January, 2004 and 4 January, 2014. Each index curve is dyed by a color code corresponding to the average change of search volumes for the respective companies of each sector *S*. The average change for each week over all company search queries for sector *S* is given by $\langle \Delta n_{S}\rangle \left(t,\;\Delta t\right)=\sum_{i}^{s}\Delta n_{i}\left(t,\;\Delta t\right)/S$, with Δ*t* being 3 weeks.

Utilizing sectoral information of each stock underlines the negative relationship of searches and markets but differs substantially across industries, as shown in Fig 2. The largest attention shifts take place with regard to financial corporations during the Subprime crisis. Additionally, the company-level data reveal some more specific intersections with real world events. Within the Materials sector, for instance, search queries see large changes in 2004 and 2005 due to regulatory inspections about DuPont´s involvement in the release of perfluorooctanoic acid (PFOA, also known as C8) into drinking water [44].

Despite these peaks in public attention, Figs 1 and 2 provide only “weak” (i.e. descriptive) evidence. Using a simple regression model with a basic control variable in terms of the S&P 500 volatility index reveals that the statistical relationship between the development of the SPY index and the average change over all (and sectoral) company search queries is indeed negative, as expected, but not significant (Table 1). This non-significance is true on a general level and for each considered sector. Hence, during regular market developments, and with it the better part of the observation period, no big changes exist in the public interest for large corporations; only through extraordinary events people seem to modify their search behavior in this respect.

**Table 1:** Influence of company search queries on the S&P 100 index development.

	〈Δ*n*〉(*t*,Δ*t*)		*VIX*_*t*_		
Sectors	Beta^a^	P-value^a^	Beta^a^	P-value^a^	R^2^ ^a^
All sectors	-0.496	0.322	-5.282	0.001	0.359
Consumer Discretionary	-0.080	0.907	-5.276	0.001	0.358
Consumer Staples	-0.080	0.822	-5.274	0.001	0.358
Energy	-0.275	0.380	-5.241	0.001	0.359
Financials	-0.172	0.378	-5.275	0.001	0.359
Health Care	-0.101	0.666	-5.282	0.001	0.358
Industrials	-0.472	0.302	-5.289	0.001	0.359
Information Technology	-0.766	0.171	-5.280	0.001	0.360
Materials	-0.077	0.620	-5.278	0.001	0.358
Telecommunication Services	0.677	0.126	-5.292	0.001	0.361
Utilities	-0.136	0.477	-5.284	0.001	0.359

^a^ Beta-coefficients, p-values and R^2^ were calculated by using a simple time-lagged regression model *SPY*_*t*+1_ = *β*_0*t*_ + *β*_1*t*_ 〈Δ*n*〉(*t*,Δ*t*)+ *β*_2*t*_ *VIX*_*t*_ to investigate the correlation of the S&P 100 index development in the next period (*SPY*_*t*+1_) with the current change over all (and sectoral) company search queries 〈Δ*n*〉(*t*,Δ*t*) and, as a basic control variable, with the volatility index of the S&P 500 (*VIX*_*t*_), respectively.

Although there is no “hard” evidence for a general connection between the average (resp. sectoral) shift in search queries and the SPY index development, peak times of financial turmoil are visibly accompanied by increased collective attention. The main part of this paper tries to exploit these collective attention shifts and investigate their relationship with individual stock prices. For this purpose, we implement a hypothetical trading strategy based on company-level Google Trends scores. The intuition behind our strategy is to take an investment position that utilizes collective attention as an indicator for “bad news” and treats an increase in collective search queries as a signal to go “short”, as it was successfully done in [33,34]. Following this approach, we first set all portfolios to an arbitrary value of 1. We implement the proposed strategy by selling a certain stock *i* at closing price *p*_*i*_(*t*) on the first trading day of week *t* and buying it back at closing price *p*_*i*_(*t* + 1) of the first trading day of the consecutive week, if the relative change in search queries is higher than the weekly average (i.e. Δ*n*_*i*_(t– 1,Δ*t*)>0). The cumulative return *R*_i_ of this “short position” is then changing by log(*p*_*i*_(*t*)) − log(*p*_*i*_(*t*+1)) If, in contrast,Δ*n*_*i*_(t– 1,Δ*t*)<0, the relative change in search volume indicates no “bad news”, but neither an immediate incentive to buy stock *i* regarding to changes in collective attention. In this case, we rely on the SPY index as a general indicator for collective financial behavior. We identify shifts in SPY prices by calculating relative changes as shown above with Google Trend scores. Thus, the “long position” is taken if the SPY index at the beginning of a trading week is higher than its average over three preceding weeks (i.e. Δ*n*_*spy*_(t– 1,Δ*t*)>0). The cumulative return *R*_*i*_ changes then by log(*p*_*i*_(*t*+1)) − log(*p*_*i*_(*t*)). If the index is below average at the beginning of a week we are going “short”. As before, the cumulative return *R*_*i*_ of this “short position” is then aggregated by log(*p*_*i*_(*t*)) − log(*p*_*i*_(*t*+1)) In summary, our investment strategy utilizes, on the one hand, Google Trends as an indicator to “short-sell” certain stocks with high attention scores, which appear especially around large market movements. On the other hand, our strategy follows the SPY index development and its mapping of general financial behavior.

**Fig 3:**
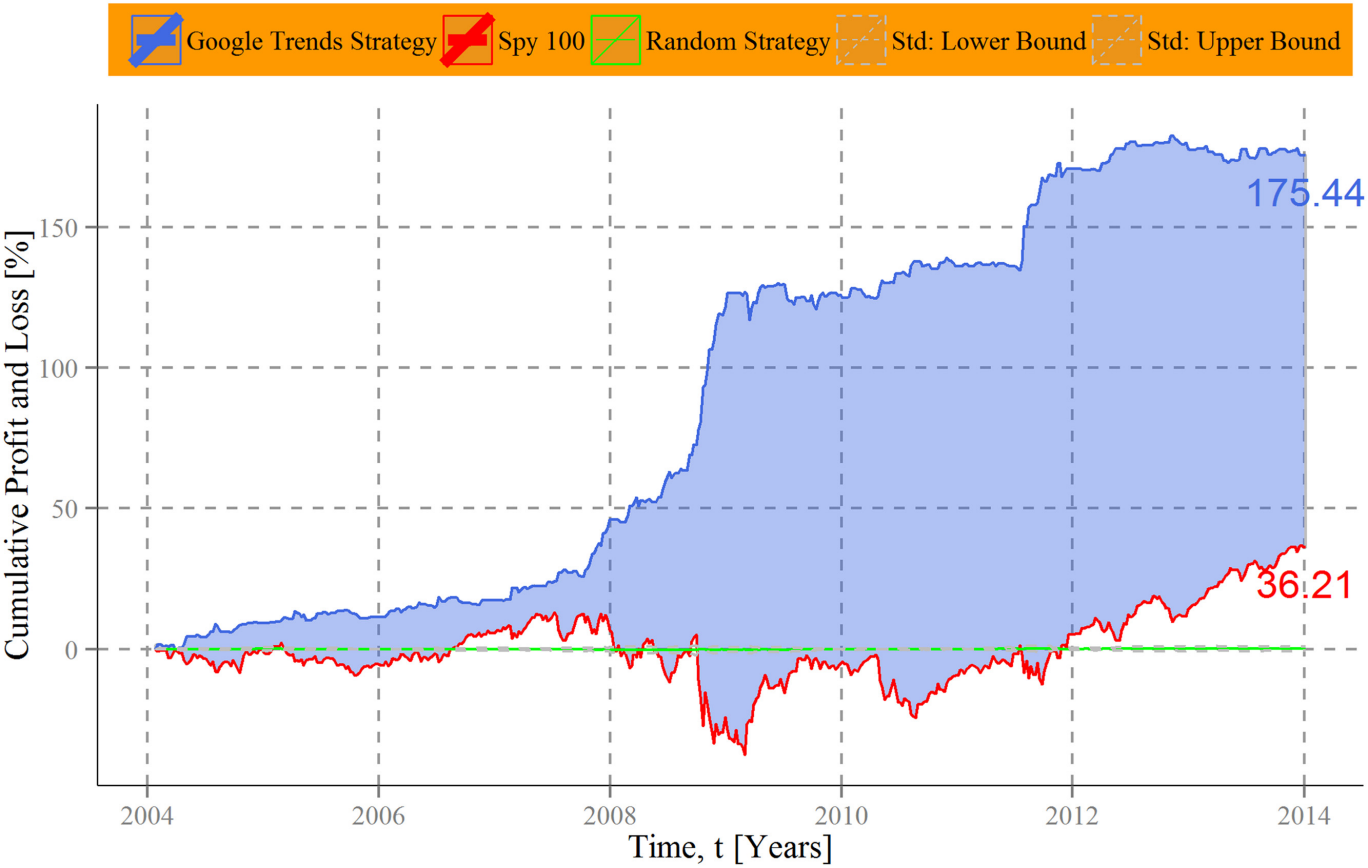
Cumulative performance of an investment strategy based on company-level Google Trends data. Cumulative profits and losses of an investment strategy based on collective attention shifts are plotted over time (blue line). The strategy utilizes company-level Google Trends data as an indicator for “bad news” and the movement of the Standard and Poor´s 100 index as an approximation of general financial behavior. To compare its performance, the cumulative return of a Standard and Poor´s 100 based “buy and hold” strategy is depicted as a red line. The difference between both lines is dyed blue. As a baseline measure we draw the mean of a random investment strategy (green line) and its standard deviation (dashed lines). The proposed investment strategy based on collective attention shifts yields a cumulative profit of circa 175%, compared to around 36% gained by Standard and Poor´s 100 index between 4 January, 2004 and 4 January, 2014. It outperforms the well-established index for the entire observation period.

In Fig 3, the mean performance of the Google Trends strategy for all stocks contained in the Standard & Poor´s 100 is illustrated by a blue line. Each company has thereby the same weight in the constructed portfolio. To get an approximation of the average market evolution we depicted the development of the index itself as a red line. Their difference is dyed blue. As baseline we implement a random strategy, in which investment decisions are generated in an uncorrelated manner by buying and selling the SPY index randomly. This simulation was executed 10,000 times and the reported results are the mean for each point in time of these procedure. The dashed lines indicate the standard deviation of the simulations. Applied for all stocks of the SPY index in the period between 2004 and 2014, the accumulated return of the Google Trends strategy is over 175%. This (hypothetical) profit outperforms the average market development indicated by the SPY index considerably, particularly in times of crisis. During financial turmoil in 2008 and 2009 the Google Trends strategy generated large profits in an extremely negative market environment. The US subprime crisis shifted mass attention—and with it Google search volumes—in the direction of certain companies. Our strategy utilizes this mechanism by “short-selling” such stocks. Otherwise it relies on general market trends, i.e. following general financial behavior. Combining both collective attention indicators, the strategy outperforms the SPY by circa 140%. Note hereby that the SPY index is already an exclusive aggregation of high-performance shares.

**Fig 4:**
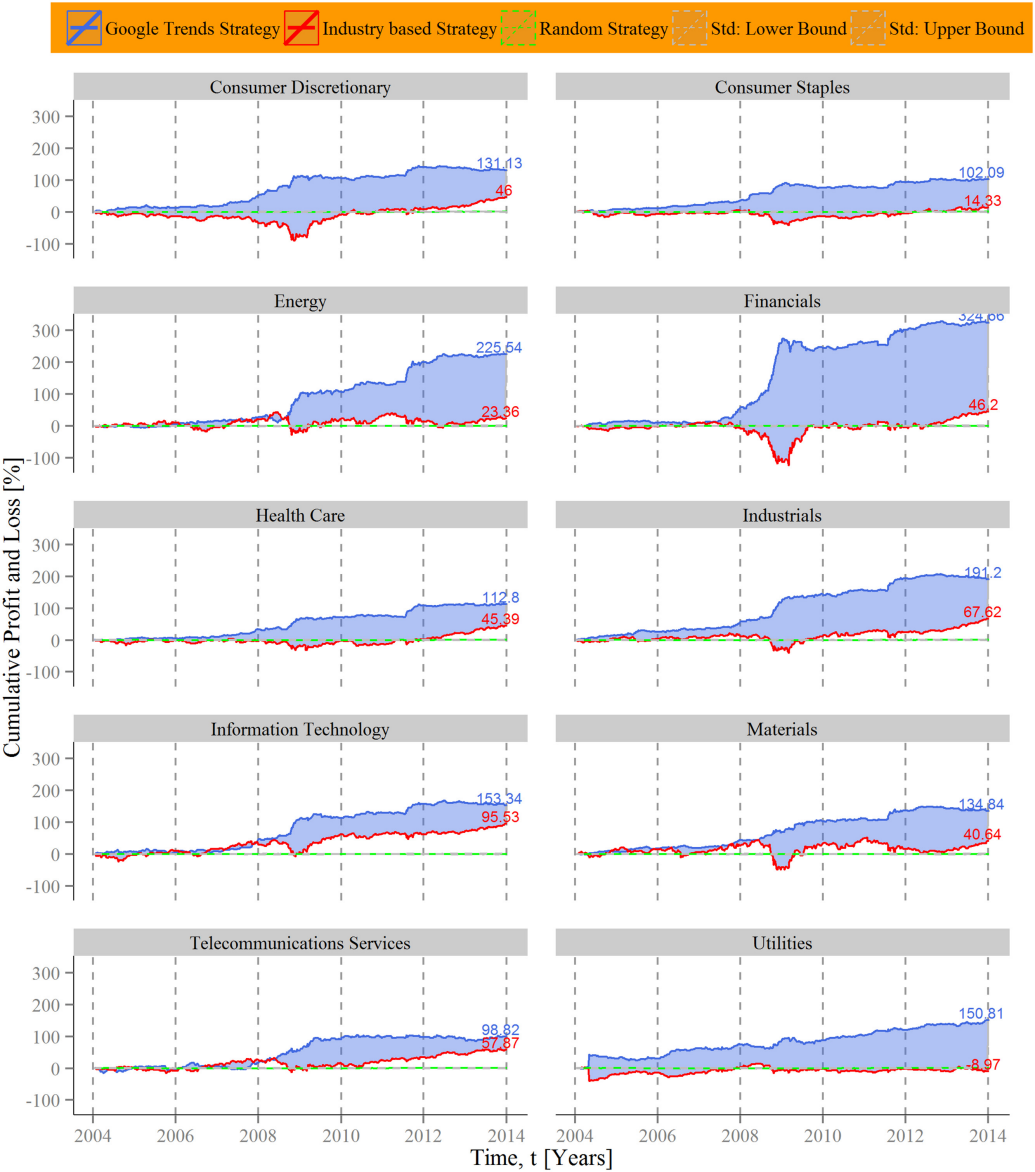
Sectoral disaggregation of cumulative returns of an investment strategy based on company-level Google Trends data. For each sector of the Standard and Poor´s 100, cumulative profit and loss of a Google Trends based investment strategy is depicted as a function of time (blue line). The hypothetical strategy aggregates company-level Google Trends data for 10 sectors and utilizes search volumes above a three weekly average as a “bad news” indicator combined with the movement of the Standard and Poor´s 100 index as an approximation of general financial behavior. To compare each sectoral performance we delineate the average of a “buy and hold” strategy based on the stocks for each industry (red line). The difference is dyed blue. A random investment strategy (green line) and its standard deviation (dashed lines) illustrate performance baselines. The highest profits could have been gained with stocks from the Financial sector (circa 325%), the lowest profits would have been realized within Telecommunication Services (around 99%). Nevertheless, for each sector the proposed investment strategy outperforms the average sectoral development between 4 January, 2004 and 4 January, 2014.

Moreover, the company-level data allows us to examine the Google Trends strategy for each of the 10 sectors included in the Standard & Poor´s 100. These results are shown in Fig 4. To compare our hypothetical investment strategy we implement a “buy and hold” strategy based on the stocks of each sector to get an “Industry based strategy” by buying all shares of an industry in the beginning and selling it at the end of each week. The mean cumulative returns of all stocks of one sector are used as sector specific baseline and depicted as red lines. Fig 4 draws a more differentiated picture of the performance of the Google Trends strategy, whereby it outperforms in every sector the industry average. However, for some sectors the Google Trends strategy produces significant higher returns than in others. Clearly, the most profound attention shifts occur in Financials. The strategy increases the value of a hypothetical portfolio that trades financial stocks over 320%. For branches relatively unaffected by the financial crisis (e.g. Health Care or Telecommunication Services) our Google Trends strategy generates lower profits, yet, still considerable higher gains than the average of the subsequent industries. This cross-sectoral pattern is consistent with the above reasoning that collective attention shifts are a major influence factor of stock price formations, since the strategy performs particularly well in times of large market movements and with companies that are in the center of massive public interest in the period between 2004 and 2014.

**Fig 5:**
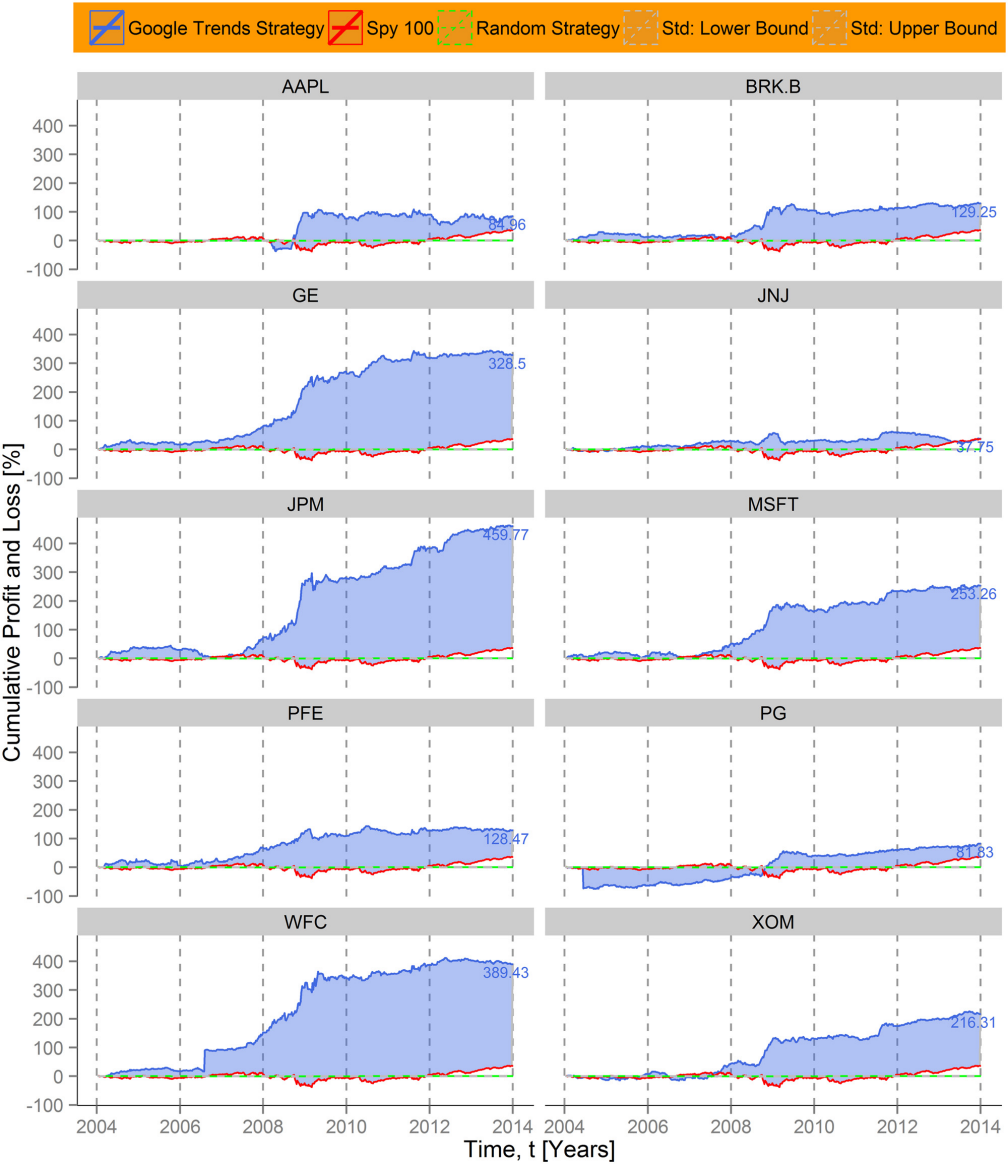
Individual cumulative returns of an investment strategy based on company-level Google Trends data. Cumulative profit and loss for each of the 10 highest weighted corporations in the Standard and Poor´s 100 index, based on a Google Trends investment strategy (blue line). To compare each performance, the average index development (~36% between 2004 and 2014) is depicted as a red line. The difference is dyed blue. A random investment strategy (green line) and its standard deviation (dashed lines) illustrate performance baselines. The results range from very high profits in the case of banks and other financial institutions (e.g. JP Morgan Chase) to rather low gains (e.g. Procter & Gamble), which highlights the eligibility of the proposed investment strategy during market upheavals.

Finally, we can investigate the effect of the proposed Google Trends strategy for individual companies. In Fig 5 we present the performance of the 10 highest weighted constituents of the Standard & Poor´s 100 [44]. Generally, the same pattern of success is visible. For all companies the Google Trend based strategy generated considerable gains, and in all but one cases (Johnson & Johnson) the cumulative profits were higher than those 36% of the index. Similar to the observations on the sectoral level, the results are related with the way of doing business and the industry affiliation, respectively. The companies benefiting most from the investment strategy are banks and other financial institutions (e.g. JP Morgan Chase), since they were in the center of the financial turmoil during the Subprime crisis. In contrast, firms with businesses that are not directly connected with the financial market (e.g. Procter & Gamble), are less eligible for the investment strategy. Again, collective attention shifts as a negative trading signal apply especially well for corporations that were hit hard by recent financial crisis.

## Discussion

Our results demonstrate that publicly available data on collective information gathering behavior can help investors in times of financial turmoil to hedge portfolio values and even extend their profits. In the period between January 2004 and January 2014 we investigated Google Trends data for companies listed in the Standard & Poor´s 100 index and detected increases in search volumes during the large market slumps in 2008 and, to a lower degree, in 2010. Investors who would have utilized these company-level search queries as an indicator for “bad news” could have gained considerable profits, particularly for sectors that were in the center of the Subprime crisis.

Interpreting Google Trends as an approximation for collective informational needs, the results may represent a general pattern in modern investment decision making regarding the importance of collective attention shifts for stock price formations. In times of large changes and great uncertainty the necessity to collect information about investment assets like stocks seems to be especially high [12,15,17,18,33,34]. In today´s world this means to “google” such companies one is interested in. At the same time, economic issues see a rise in media coverage during such market upheavals [13] and, hence, are more likely incorporated into everyday-life conversation topics. This (and many more conceivable) micro social processes become manifest in increasing search volumes for affected companies and are succeeded by decreasing stock prices. Therefore, changes in collective attention are an important reason for the particular success of our trading strategy during recent crisis and within afflicted sectors and companies; an explanation that follows directly Herbert Simon´s suggestion about attention as a scarce commodity which enhances the gathering of information especially in times of economic uncertainty [36].

However, the pattern applies particularly well if the state of the economy is turning drastically [20]. This means collective attention in terms of Google Trends data serve especially well as an indicator for “*bad* news” and subsequent falling prices, which can be exploited by going “short” for those assets. In contrast, people seem not inclined to search in great numbers for corporations that are presenting, for instance, respectable annual reports or announce noteworthy sales numbers. Thus, collective attention for large companies may follow the general media logic that “good news is no news”, or in our case more precisely, that “only bad news is relevant news” in order to use it as an investment signal. In this way, the interplay between collective information gathering and financial behavior may even contribute to the overreaction of investors during financial crises and the subsequent magnification of economic slumps.

As a consequence, Google Trends data offer mainly a possibility to investigate collective financial behavior and search queries in *negative* economic contexts. Instead, regular market environments with steadily rising prices seem less connected to collective attention shifts, as far as current evidence tells us. Nevertheless, we are convinced that in the near future more disaggregated data will be available, so that *regular* market contexts can also be investigated in greater depths and advance our knowledge about complex social systems.

## Material

We retrieved search volume data from Google Trends website (http://www.google.com/trends) on every day between August 23, 2014 and August 29, 2014 for all companies in the Standard and Poor´s 100 index. The index composition is taken as of August 29, 2014 and has not changed since then. Search volume data are restricted to requests of users localized in the USA, the home location of all companies contained in the Standard and Poor´s 100 index. The series are reported weekly on a Sunday to Saturday frequency. Since only five search terms can be looked up simultaneously, we retrieved the data for each company separately. To avoid semantic ambiguities we used the full company name plus the word “company”. The search volumes are normalized by Google with a maximum of 100, serving as a scaling factor for the rest of the series. Due to this normalization Google Trends results are dependent from the time of observation. Therefore, we provide in S1 File the original data used in this article to facilitate the reproduction of our results.

**Fig 6:**
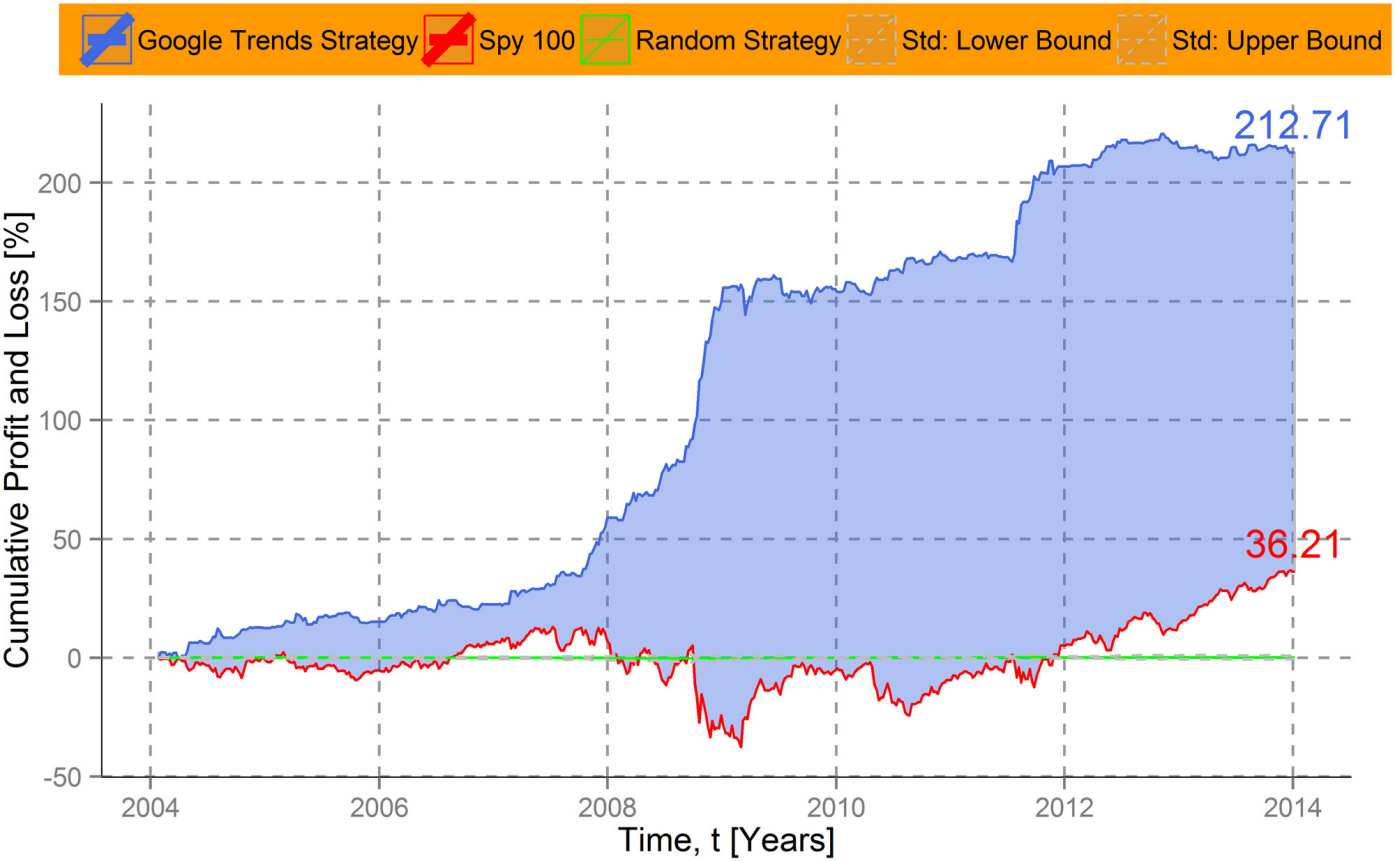
Cumulative returns of an investment strategy based on company-level Google Trends data for 71 corporations of the Standard and Poor´s 100. Cumulative profit and loss for all corporations in the dataset that have no more than 4 missing values over the whole observation period between 4 January, 2004 and 4 January, 2014. Profits and losses are based on a Google Trends investment strategy (blue line). The development of the curve is very similar to the results displayed in Fig 3, which uses all available data. However, using only the 71 corporations that meet the stricter condition increases the overall profits.

However, there are missing values of different degrees within the dataset. For five companies we could not find any Google Trends scores with the described search terms. They have no values for all 522 weeks and are therefore not included in the analysis. A detailed distribution of the missing values can be found in S2 File. For 71 companies we have “complete” data in the sense that not more than four weeks are missing for the whole observation period. Calculating the results only for those companies, Fig 6 shows a very similar development and increases the performance of the proposed investment strategy slightly. Thus, the results for the entire dataset represent a lower boundary for possible profits, i.e. that stricter data cleaning can even improve profits.

To further support the reliability of the proposed trading strategy, we calculated another possible approach: We reversed the whole trading strategy by *buying* (instead of selling) if search volumes are above average. If this case is not applying, we are trusting the SPY index if its *below* its mean (i.e. Δ*n*_*spy*_(t– 1,Δ*t*)<0) and are selling otherwise, which is for both elements of the strategy the exact opposite as initially suggested. The subsequent results are shown in S4 File and represent the inversion of the curve presented in Fig 3. Thus, if an investor would have applied this (hypothetical) trading strategy and used Google Trends scores as a buying signal, she would have generated a huge loss.

Furthermore, the stock prices were downloaded from Yahoo Finance (http://finance.yahoo.com) on a daily basis for every trading day between January 4, 2004 and January 4, 2014. Closing prices *p*_*i*_(*t*) at the first trading day of a week are matched to Google Trends data of the previous week, when information would be available for hypothetical investors. In a regular trading week, for instance, closing price *p*_*i*_(*t*) on Monday would correspond to Google search queries for *i* in the previous week, which is available on the preceding Saturday.

All steps of the analysis described above were conducted with the open-source language Python. All Figures were drawn with the R-Package “ggplot2”.

## Supporting Information

S1 File Google Trends data for Standard and Poor´s 100 companies. (ZIP)

S2 File Missing values of the Google Trends data for Standard and Poor´s 100 companies. (CSV)

S3 File Different time windows for the proposed Google Trends investment strategy. (TIF)

S4 File Cumulative performance of a strategy that reverses the proposed investment mechanism. The reversed trading strategy is buying (instead of selling) if search volumes are above average and trusting the SPY index if its below its mean and selling otherwise, which is for both elements the opposite as suggested in Fig 3.
(TIF)
