# Supplementary figures and images for: Collective Attention and Stock Prices: Evidence from Google Trends Data on Standard and Poor's 100
Source: PLoS One. 2015 Aug 10;10(8):e0135311. doi: 10.1371/journal.pone.0135311 (PMC4530949; doi:10.1371/journal.pone.0135311)

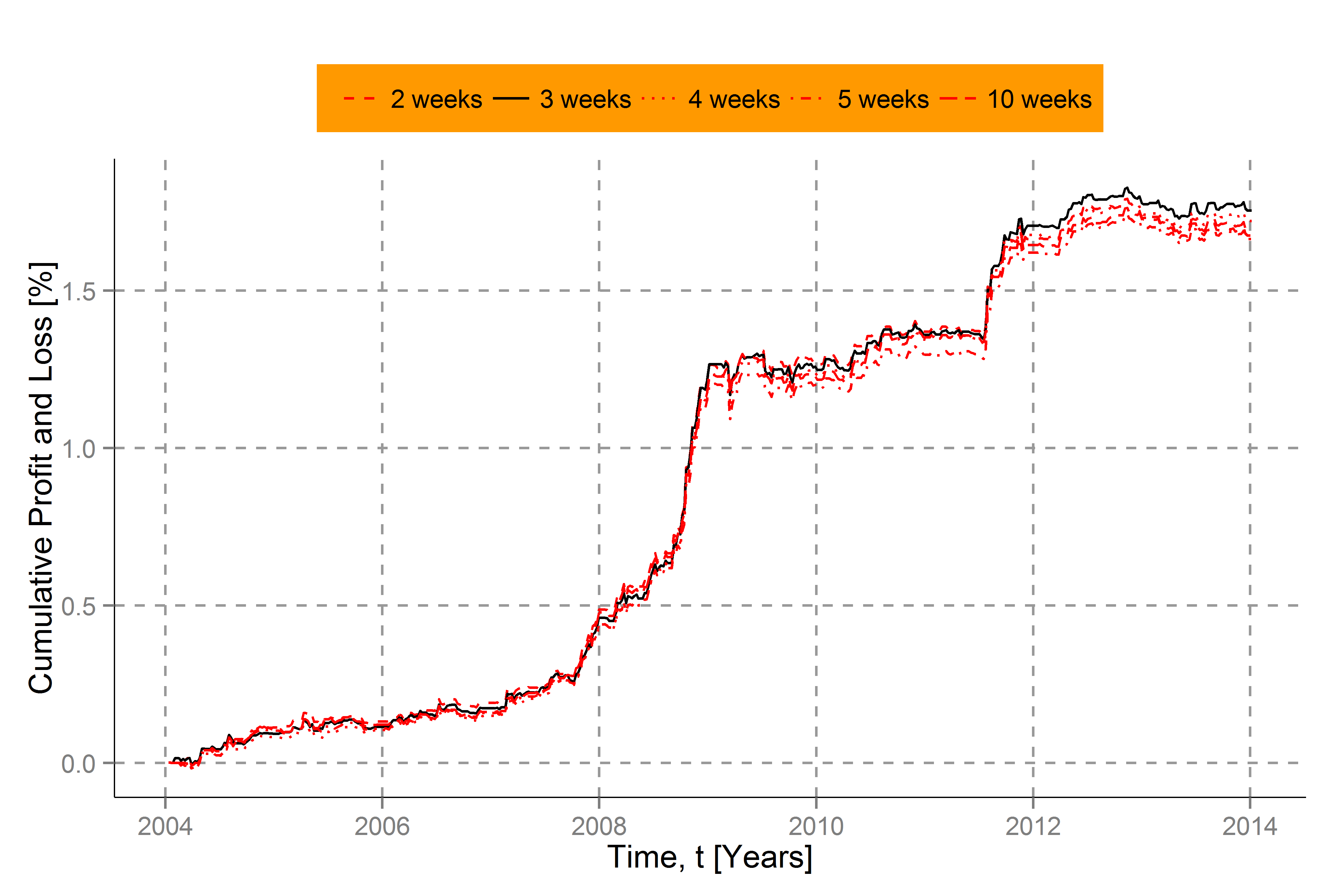

Supplement: S3 File — (TIF) [file pone.0135311.s003.tif]

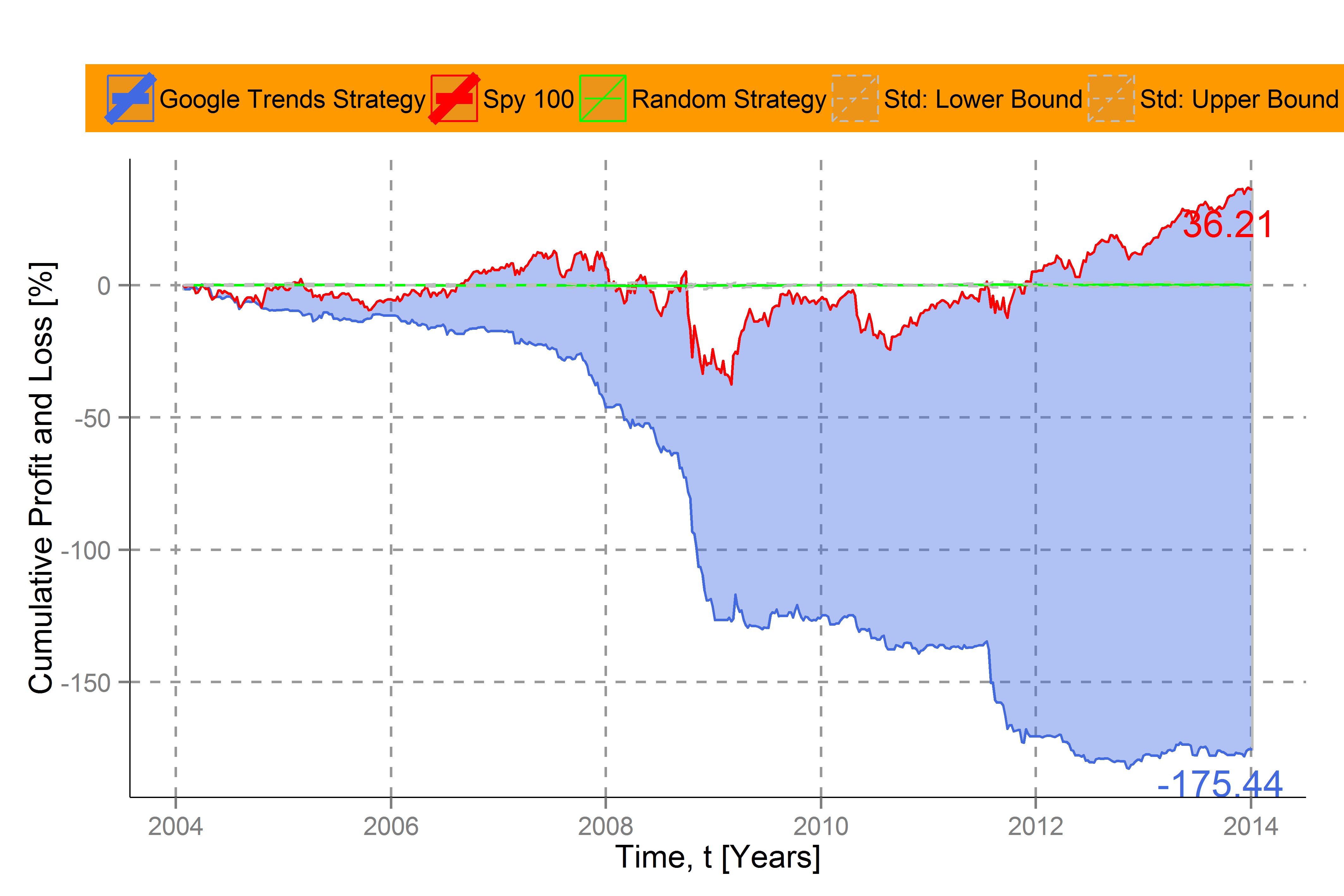

Supplement: S4 File — The reversed trading strategy is buying (instead of selling) if search volumes are above average and trusting the SPY index if its below its mean and selling otherwise, which is for both elements the opposite as suggested in Fig 3. (TIF) [file pone.0135311.s004.tif]
